# Supplementary material for: Dynamic Greenland ice sheet driven by pCO2 variations across the Pliocene Pleistocene transition
Source: Nat Commun. 2018 Nov 12;9:4755. doi: 10.1038/s41467-018-07206-w (PMC6232173; doi:10.1038/s41467-018-07206-w)
Supplement: Supplementary file 1 — Supplementary Information [file 41467_2018_7206_MOESM1_ESM.pdf]

Supplementary Information

Dynamic Greenland ice sheet driven by pCO_2 variations across the Pliocene Pleistocene transition

Tan et al.

Supplementary Note 1. IRD surrounding Greenland available for model comparison

The available IRDs used in this study are obtained respectively from ocean drilling project (ODP) site 907 [1], ODP site 611 [2] and Integrated Ocean Drilling Project (IODP) site U1307 [3].

It is important to note that the available IRD datasets differ in temporal resolution, in the method of IRD detection, and the size fraction of mineral grains. The IRD peaks that occur during the PPT are indicative of the presence of dynamic glacial margins. The peaks present in these datasets are general smaller than those observed in Late Pleistocene IRD events which have been considered to indicate the occurrence of large-scale ice sheet collapses [4,5]. They do however represent significant influx of mineral grains from terrestrial provenances into deep marine settings, which in turn indicate the presence glacial margin dynamics. The specific definition of IRD used in the studies references here varies in some details. For ODP Site 907, Jansen et al [1] counted all terrestrial non-volcanic mineral grains >125 μm as IRD, giving IRD concentration data for their samples. For Site 611, Bailey et al [2] counted mineral grains >150 μm in size and provide both IRD concentration and flux rate, which are largely in agreement with each other (i.e. there is no significant effect of sedimentation rate on the IRD variability). For IODP Site U1307, Sarnthein et al [6] do not provide a clear definition of IRD, simply displaying concentrations. The fact that, despite these differences in IRD definition as well as the different regional settings, significant increases in IRD coincide at ~2.7 Ma, gives a clear indication of increased glacial margin dynamics.

According to [7] an important difference between PPT and more recent (Late Pleistocene) ice rafting may play a role in absolute IRD abundance differences as well. Modern icebergs are relatively clean, containing only small amounts of terrestrial mineral grains, which means that they would provide only small amounts of IRD. Early expansions of the GrIS during the PPT could potentially have contained more mineral grains per iceberg. Bailey et al [2] discuss a possible different type of ice sheet behaviour (movement of ice sheets over land) during the Pliocene-Pleistocene transition compared to that of large (e.g. modern Greenland) ice

sheets, which could potentially lead to changes in IRD signals without large-scale changes in the GrIS volume in the Late Pliocene / Early Pleistocene.

The available SST records used in this study are obtained respectively from ODP site 982 [8] and from re-drill of Deep Sea Drilling Project (DSDP) site 607 [9]. These data are obtained by using the alkenone unsaturation index ($U_{37}^{k`}$) and show the reconstructed mean annual temperatures at the surface. The referenced SST time series are resolved at orbital time scales (between 2 ka and 4 ka). Other details about these data can be found in the given references. The location of all these sites is shown in Fig.5a.

Supplementary Table 1. Forcing factors of reference AOGCM experiments.

Orbital configuration	pCO_2 (ppmv)	Vegetation settings	Prescribed GrIS size
Cold orbit (2.601 Ma)<br>Or<br>Warm orbit (2.589 Ma)	220	**cold orbit:** PlioMIP modified [a]	S0, S1…S6
		**Warm orbit:** PlioMIP	
	280	**cold orbit:** PlioMIP modified [a]	S0, S1…S6
		**Warm orbit:** PlioMIP	
	360	**cold orbit:**PlioMIP modified [b]	S0, S1…S6
		**Warm orbit:**PlioMIP	
	405	**cold orbit:**PlioMIP modified [b]	S0, S1…S6
		**Warm orbit:** PlioMIP	

a. tundra north of 50°N; b. tundra north of 65°N.

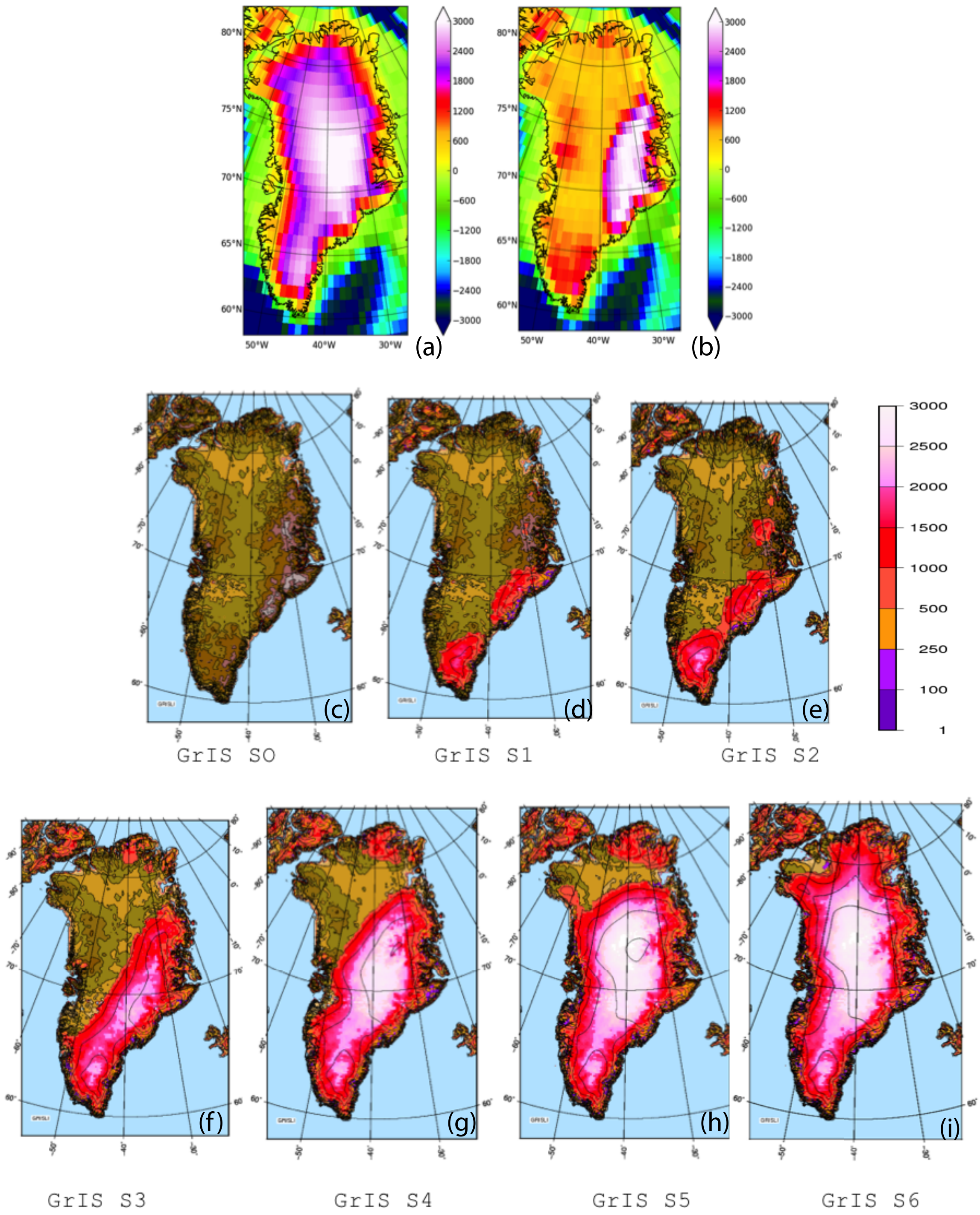

Supplementary Figure 1. Greenland ice sheet configurations. (a) and (b) present respectively the Pre-industrial and the reconstructed PlioMIP (~3.2 Ma) [10] Greenland topography (m). (c), (d), (e), (f), (g), (h), (i) present the seven simulated GrIS scenarios in this study which are imposed in the AOGCM experiments.

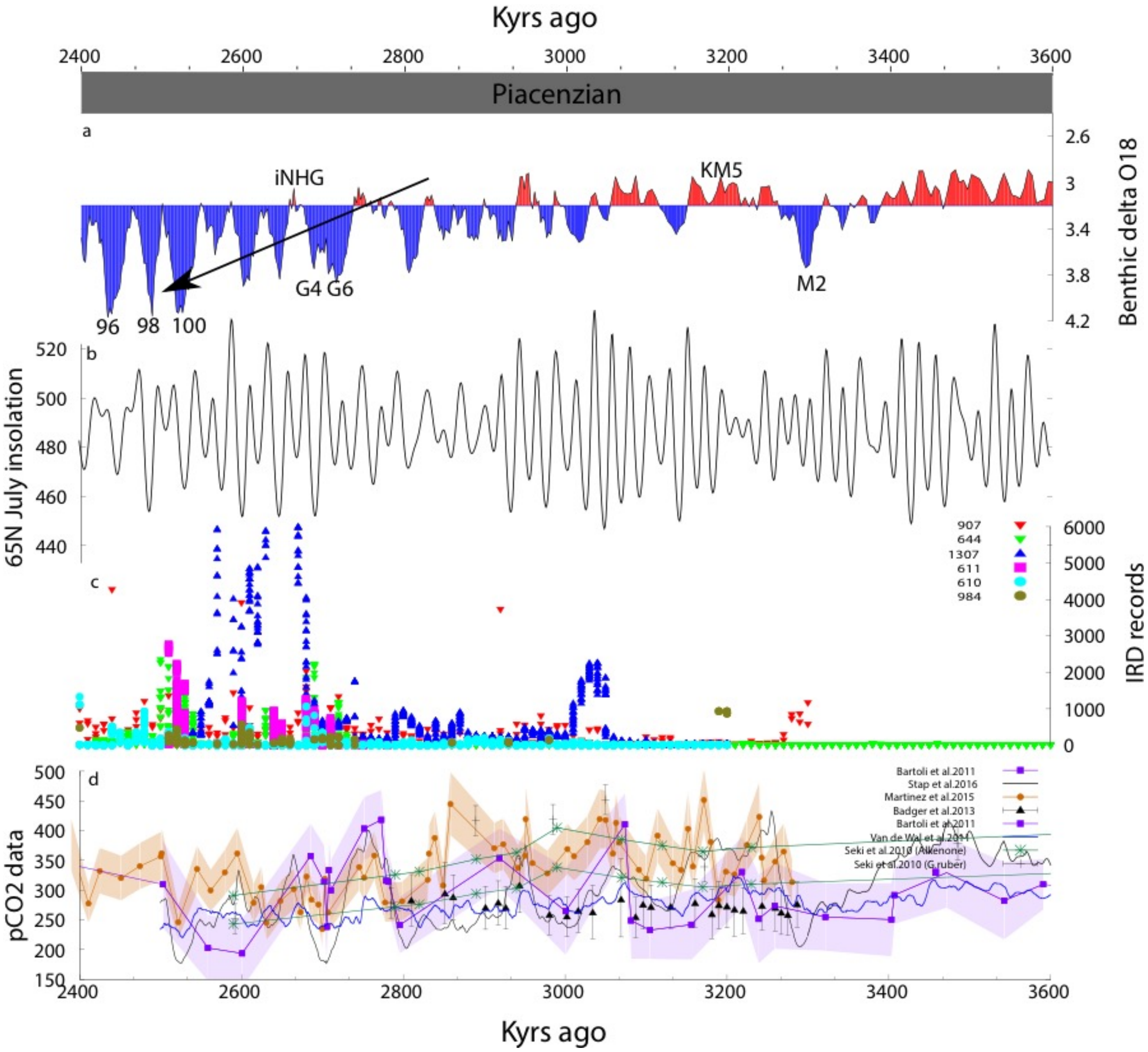

Supplementary Figure 2. A synthesis of Late Pliocene evolution. (a) LR04 benthic isotope stack [11] ; (b) July insolation at 65N [12]; (c) Ice rafted detritus (IRD) records from different studies (DSDP Site 610 [13], DSDP Site 611 [2], ODP Site 644 [14], ODP Site 907 [1], ODP Site 984 [15] and site U1307 [6]); (d) Reconstructed pCO_2 records and model inverse data from different studies [16-21]. pCO_2 data from Stap et al [21] (black line) and Van de Wal et al [20] (blue line) are obtained with the model inverse method. pCO_2 records from Martinez et al [19] (brown circles), Badger et al [18] (black triangles), Bartoli et al [17] (purple squares) and Seki et al [16] (green asterisks) are alkenone-based records. The envelope of Martinez et al [19] encompasses 68% of 10,000 Monte Carlo simulations of pCO_2. Error bars of Badger et al [18] show a full Monte Carlo propagation of associated

uncertainties. The envelope of Bartoli et al [17] shows the uncertainty range of 2-sigma from the average. Upper and lower alkenone-based pCO_2 estimates from Seki et al [16] were obtained by using two different estimations of alkalinity. The error bars of δ11B-based pCO_2 records from Seki et al [16] (black crosses) are +-25 ppmv from the average.

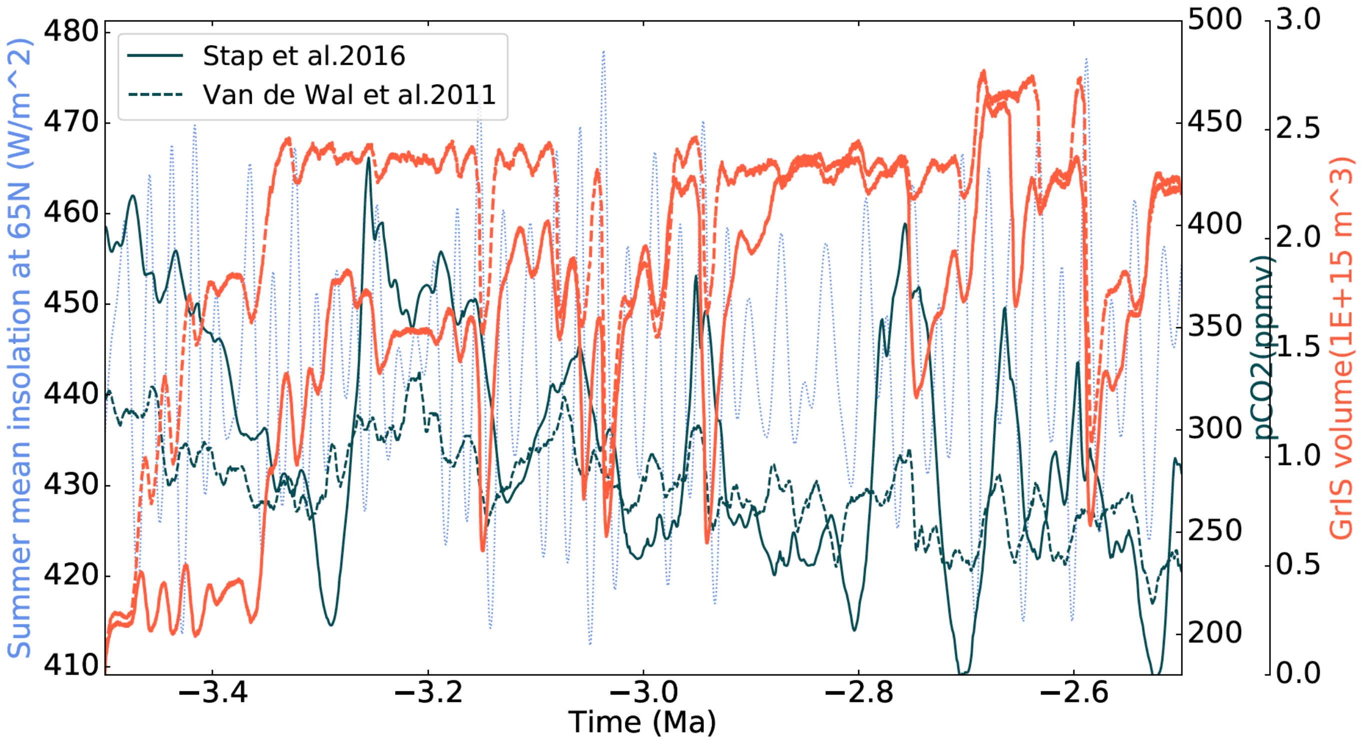

Supplementary Figure 3. Simulated GrIS volumes during 3.5-2.5 Ma based on two sets of model inverse pCO2 data [19,21] . Light blue dash line represents the boreal summer insolation at 65N, Teal and orange dash lines present respectively the pCO2 data from van de Wal et al [20] and the associated simulated GrIS volume. Green and orange solid lines present respectively the pCO2 data from Stap et al [21] and the associated simulated GrIS volume.

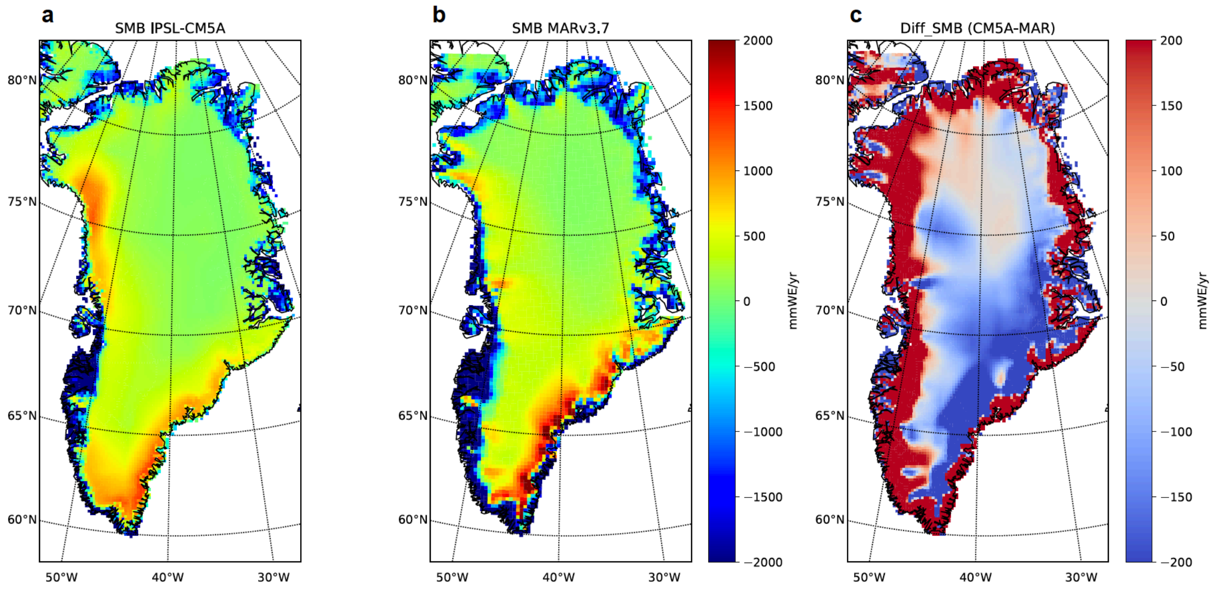

Supplementary Figure 4. Simulated surface mass balance (SMB) for the averaged last 25 years (1981-2005) (units in mmWE/yr). (a) SMB from IPSL-CM5A-LR AOGCM model, (b)SMB from MAR regional model, (c) SMB difference between IPSL-CM5A-LR AOGCM model and MAR regional model.

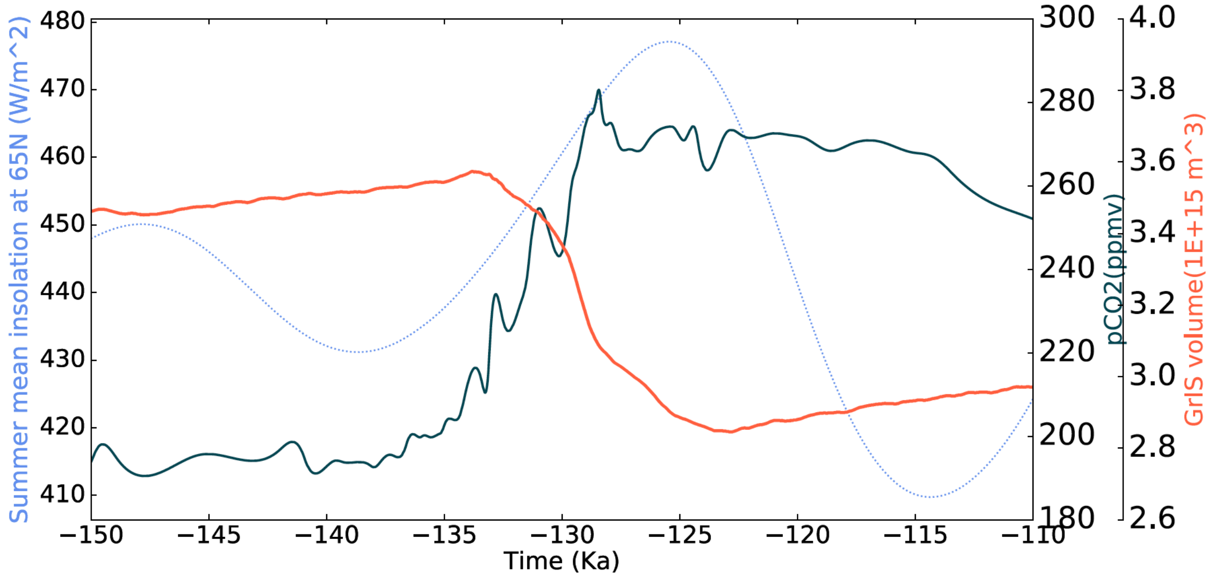

Supplementary Figure 5. The simulated GrIS volume based on the pCO2 records from 150Ka to 110 Ka. The blue line represents the summer insolation at 65N[12]. The green teal line represents the pCO2 records [22], the orange line is the simulated GrIS in this study.

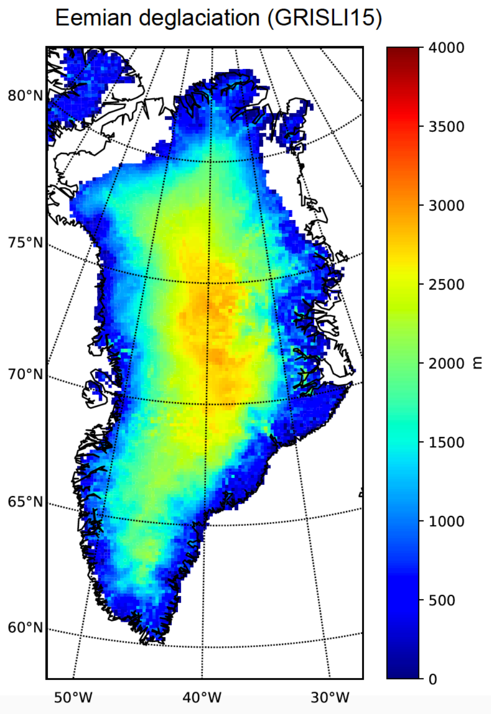

Supplementary Figure 6. The simulated GrIS thickness during the Eemain deglaciation around 123Ka.

Supplementary References

1. Jansen, E., Fronval, T., Rack, F. & Channell, J. E. T. Pliocene-Pleistocene ice rafting history and cyclicity in the Nordic Seas during the last 3.5 Myr. *Paleoceanography* **15,** 709–721 (2000).

2. Bailey, I. *et al*. An alternative suggestion for the Pliocene onset of major northern hemisphere glaciation based on the geochemical provenance of North Atlantic Ocean ice-rafted debris. *Quat. Sci. Rev*. **75,** (2013).

3. Sarnthein, M., Prange, M., Schmittner, a., Schneider, B. & Weinelt, M. Mid-Pliocene shifts in ocean overturning circulation and the onset of Quaternary-style climates*. *Clim. Past Discuss*. **5,** 251–285 (2009).

4. Heinrich, H. Origin and consequences of cyclic ice rafting in the northeast Atlantic Ocean during the past 130,000 years. *Quat. Res*. **29,** 142–152 (1988).

5. Bond, G. C. & Lotti, R. Iceberg discharges into the North Atlantic on millennial time scales during the last glaciation. *Science (80-.)*. **267,** 1005–1010 (1995).

6. Sarnthein, M., Prange, M., Schmittner, a., Schneider, B. & Weinelt, M. Mid-Pliocene shifts in ocean overturning circulation and the onset of Quaternary-style climates*. *Clim. Past Discuss*. **5,** 251–285 (2009).

7. Andrews, J. T. Icebergs and iceberg rafted detritus (IRD) in the North Atlantic: facts and assumptions. *Oceanography* 100–108 (2000).

8. Lawrence, K. T., Herbert, T. D., Brown, C. M., Raymo, M. E. & Haywood, A. M. High-amplitude variations in north atlantic sea surface temperature during the early pliocene warm period. *Paleoceanography* **24,** 1–15 (2009).

9. Naafs, B. D. A. *et al.* Late Pliocene changes in the North Atlantic Current. *Earth Planet. Sci. Lett.* **298,** 434–442 (2010).

10. Haywood, a. M. *et al.* Pliocene Model Intercomparison (PlioMIP) Phase 2: scientific objectives and experimental design. *Clim. Past Discuss.* **11,** 4003–4038 (2015).

11. Lisiecki, L. E. & Raymo, M. E. A Pliocene-Pleistocene stack of 57 globally distributed benthic δ 18 O records. *Paleoceanography* **20,** n/a-n/a (2005).

12. Laskar, J. *et al.* A long-term numerical solution for the insolation quantities of the Earth. *Astron. Astrophys.* **428,** 261–285 (2004).

13. Flesche Kleiven, H., Jansen, E., Fronval, T. & Smith, T. M. Intensification of Northern Hemisphere glaciations in the circum Atlantic region (3.5-2.4 Ma) - Ice-rafted detritus evidence. *Palaeogeogr. Palaeoclimatol. Palaeoecol.* **184,** 213–223 (2002).

14. Jansen, E. & Sjøholm, J. Reconstruction of glaciation over the past 6 Myr from ice-borne deposits in the Norwegian Sea. *Nature* **349,** 600–603 (1991).

15. Bartoli, G., Sarnthein, M. & Weinelt, M. Late Pliocene millennial-scale climate variability in the northern North Atlantic prior to and after the onset of Northern Hemisphere glaciation. *Paleoceanography* **21,** (2006).

16. Seki, O. *et al.* Alkenone and boron-based Pliocene pCO2 records. *Earth Planet. Sci. Lett.* **292,** 201–211 (2010).

17. Bartoli, G., Hönisch, B. & Zeebe, R. E. Atmospheric CO2 decline during the Pliocene intensification of Northern Hemisphere glaciations. *Paleoceanography* **26,** (2011).

18. Badger, M. P. S. Schmidt, D., Mackensen, A., & D Pancost, R. High-resolution alkenone palaeobarometry indicates relatively stable pCO2 during the Pliocene (3.3-2.8 Ma). *Philosophical Transactions. Series A, Mathematical, Physical, and Engineering Sciences*, *371*, 20130094.(2013).

19. Martínez-Botí, M. a. *et al.* Plio-Pleistocene climate sensitivity evaluated using high-resolution CO2 records. *Nature* **518,** 49–54 (2015).

20. Van De Wal, R. S. W., De Boer, B., Lourens, L. J., Köhler, P. & Bintanja, R. Reconstruction of a continuous high-resolution CO 2 record over the past 20 million years. *Clim. Past* **7,** 1459–1469 (2011).

21. Stap, L. B. *et al.* CO2 over the past 5 million years: Continuous simulation and new δ11B-based proxy data. *Earth Planet. Sci. Lett.* **439,** 1–10 (2016).

22. Petit, R. J. *et al.* Climate and atmospheric history of the past 420,000 years from the Vostok ice core, Antarctica. *Nature* **399,** 429–413 (1999).
