# Supplementary material for: Dynamic Greenland ice sheet driven by pCO2 variations across the Pliocene Pleistocene transition
Source: Nat Commun. 2018 Nov 12;9:4755. doi: 10.1038/s41467-018-07206-w (PMC6232173; doi:10.1038/s41467-018-07206-w)
Supplement: Supplementary file 3 — Description of Additional Supplementary Files [file 41467_2018_7206_MOESM3_ESM.pdf]

Description of Additional Supplementary Files

Supplementary Movie 1

Description: Simulated Greenland ice sheet thickness evolution along with the variations of the mean pCO_2 estimates of Martienz-Boti et al (2015) and summer insolation (65°N) (Laskar et al., 2004) across the Plio-Pleistocene transition (3.0-2.5 Ma).
